# Distinct lung microbiota associate with HIV-associated chronic lung disease in children

**DOI:** 10.1038/s41598-020-73085-1

**Published:** 2020-09-30

**Authors:** Sudha Bhadriraju, Douglas W. Fadrosh, Meera K. Shenoy, Din L. Lin, Kole V. Lynch, Kathryn McCauley, Rashida A. Ferrand, Edith D. Majonga, Grace McHugh, Laurence Huang, Susan V. Lynch, John Z. Metcalfe

**Affiliations:** 1 Division of Pulmonary and Critical Care Medicine, San Francisco General Hospital and Trauma Center, University of California San Francisco, 1001 Potrero Avenue, Rm 5K1, San Francisco, CA 94110-0111 USA; 2 Division of Gastroenterology, Department of Medicine, University of California, San Francisco, San Francisco, USA; 3 Biomedical Research and Training Institute, Harare, Zimbabwe; 4 Clinical Research Department, London School of Hygiene and Tropical Medicine, London, UK

**Keywords:** HIV infections, Respiratory tract diseases

## Abstract

Chronic lung disease (CLD) is a common co-morbidity for HIV-positive children and adolescents on antiretroviral therapy (ART) in sub-Saharan Africa. In this population, distinct airway microbiota may differentially confer risk of CLD. In a cross-sectional study of 202 HIV-infected children aged 6–16 years in Harare, Zimbabwe, we determined the association of sputum microbiota composition (using 16S ribosomal RNA V4 gene region sequencing) with CLD defined using clinical, spirometric, or radiographic criteria. Forty-two percent of children were determined to have CLD according to our definition. Dirichlet multinomial mixtures identified four compositionally distinct sputum microbiota structures. Patients whose sputum microbiota was dominated by *Haemophilus, Moraxella or Neisseria* (*HMN*) were at 1.5 times higher risk of CLD than those with *Streptococcus* or *Prevotella* (*SP*)-dominated microbiota (RR = 1.48, p = 0.035). Cell-free products of *HMN* sputum microbiota induced features of epithelial disruption and inflammatory gene expression in vitro, indicating enhanced pathogenic potential of these CLD-associated microbiota. Thus, HIV-positive children harbor distinct sputum microbiota, with those dominated by *Haemophilus, Moraxella or Neisseria* associated with enhanced pathogenesis in vitro and clinical CLD.

## Introduction

In 2019, 1.8 million children aged less than 15 years were living with HIV, 90% in sub-Saharan Africa^1^. Global scale-up of ART has resulted in a dramatic improvement in survival, with children accessing ART surviving to adulthood^2,3^. Although acute bacterial pneumonias, disseminated *M. avium* complex, *Pneumocystis jeroveci* pneumonia, and lymphocytic interstitial pneumonitis have declined in this age group since the pre-ART era^4^, respiratory disease remains a major cause of morbidity and mortality in HIV-infected children^2^.

Chronic respiratory symptoms, including cough (21–60%), dyspnea (12–18%), and exertional hypoxemia (12–38%), are common among older HIV-positive older children^3^. Further, HIV-positive adolescents have reduced exercise tolerance and spirometric abnormalities including lower forced expiratory volume in one second (FEV1) and forced vital capacity (FVC) relative to HIV-negative controls^2,3^. Although the pathogenesis of HIV-associated chronic lung disease (CLD) remains unknown, high resolution chest computed tomography (HRCT) findings of decreased attenuation and bronchiectasis suggest small airways pathology, possibly due to recurrent pulmonary infections and HIV-mediated airway inflammation^3^.

Several risk factors have been implicated in the pathogenesis of HIV-CLD including HIV-mediated systemic immune activation and chronic inflammation^3,5^. Recent study of the lung microbiota and its mechanistic role in mediating the lung’s inflammatory response has revitalized our understanding of the pathogenesis of chronic lung diseases like asthma^6–8^. Bacterial products, ligands, and metabolites, and the host response to them can promote inflammation and increase lung susceptibility to oxidative stress, resulting in increased risk for acute respiratory infection and chronic lung disease^7^.

Our group previously demonstrated that three distinct airway microbiota exist in HIV-infected adults with pneumonia, and that these microbiota are associated with distinct airway mucosal inflammatory profiles and short-term mortality^9^. Here, we extend our work to children and adolescents with HIV on ART to examine the association of airway microbiota with clinical evidence of CLD. We hypothesized the presence of distinct sputum microbiota among children with CLD versus those without, and that in human airway epithelial cell experiments such microbiota would induce inflammation mechanistically important to CLD pathobiology.

## Results

### Study participants

We recruited a total of 202 HIV-positive children aged 6–16 years stable on ART. The overall prevalence of clinical indicators of HIV-CLD was 42.1% (n = 85). A total of seven children had abnormal spirometry, 32 had chronic cough and dyspnea, and the remainder had abnormal CT imaging. Participants with CLD were older than those without CLD (11.6 vs. 10.6 years; p < 0.01); other demographic features were similar (Table 1). Time on ART, CD4+ T-cell count, and proportion virologically suppressed did not differ between groups. More than one-third of participants (38%) had experienced one or more prior episodes of tuberculosis treatment, and this was similar among those with (41.2%) or without (35.9%) CLD. A total of 151 sputum samples were collected and processed for 16S rRNA biomarker sequencing. Participants whose sputum samples were unable to be sequenced were statistically younger (9.8 vs. 10.8 years; p = 0.01) and were younger at time of HIV diagnosis (4.4 vs. 5.3 years; p = 0.01) relative to those whose samples were processed for 16S rRNA biomarker sequencing; other demographic and clinical characteristics were similar (Supplemental Table 2).

**Table 1:** Characteristics of study participants.

	Participants without CLD (n = 117)	Participants with CLD (n = 85)	p-value
Age (years), mean (SD)	10.6 (2.4)	11.6 (2.8)	0.006*
Female sex, n (%)	54 (46.2%)	37 (43.5%)	0.71
Age at diagnosis (years), mean (SD)	4.6 (2.8)	5.8 (3.2)	0.008*
Duration of ART at recruitment (years), mean (SD)	4.8 (2.5)	4.8 (2.8)	0.93
CD4 + at enrollment (cells/μL), mean (SD)	739 (278.9)	737 (421.4)	0.97
Viral load at enrollment (copies/mL), median (IQR)^†^	19 (49)	20 (328)	0.12
Proportion virally suppressed (< 75 copies/mL)	72%	68%	0.54
Previous TB treatment^‡^	42 (35.9%)	35 (41.2%)	0.45
Treated with ≥ 1 course of antibiotics in the past 12 months^‡^	27 (23.1%)	18 (21.2%)	0.75
Stunted growth^‡§^	40 (34%)	32 (37.6%)	0.64
**Radiographic features on HRCT**^‡^			
Bronchial wall thickening	0 (0%)	69 (95.8%)	0.001*
Bronchiectasis	0 (0%)	28 (38.9%)	0.04*
Bronchiectasis and mosaic attenuation	0 (0%)	23 (31.9%)	0.07

*SD* standard deviation, *CLD* chronic lung disease, *TB* tuberculosis, *HRCT* high resolution chest computed tomography.

*p < 0.05 = significant; ^†^Wilcoxon-rank sum test; ^‡^Chi-square test or Fisher’s exact test; ^§^”Height for age" value ≤ two standard deviations of the WHO Child Growth Standards^45^.

### Determination of sputum microbiota structures

**Figure 1:**
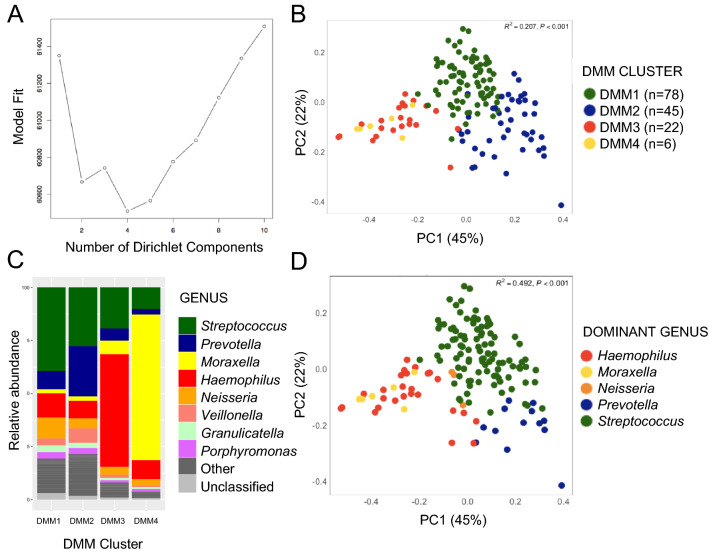
(**A**) LaPlace approximation of Dirichlet Multinomial Mixture Model fit indicating that four distinct sputum microbiota structures are evident in our pediatric cohort. (**B**) Distinct microbiota structures identified by Dirichlet multinomial mixture model (DMM) explain approximately 20% of variance in sputum microbiota composition (Weighted UniFrac). (**C**) Stacked bar graphs indicate that each DMM cluster is dominated by a distinct bacterial genus. (**D**) Distinct sputum microbiota dominated by *Prevotella*, *Streptococcus*, *Haemophilus*, *Moraxella* and *Neisseria* exist in the airways of children with HIV infection.

Sputum samples undergoing 16S rRNA biomarker sequencing attained a median of 184,171 high quality reads per sample (range = 78,309–396,824 reads), which after filtering and rarefying to 73,265 were binned into a total of 365 operational taxonomic units (OTUs). Mathematical modeling of OTU frequencies using Dirichlet multinomial mixtures (DMM) can be applied to 16S rRNA datasets to determine whether distinct microbiota structures exist and repeat across participants within a cohort. Using a Laplace approximation (Fig. 1a), DMM indicated that 4 significantly distinct bacterial sputum microbiota structures (DMM1-4) existed in the dataset. Principal components analyses confirmed that these four microbiota states (DMM1-4) explained a reasonable proportion of observed variance in sputum microbiota composition observed in our dataset (R^2^ = 0.207, p < 0.001; Fig. 1b). We next examined the distribution of taxa within each of these sputum DMM clusters and noted that each was dominated by a distinct bacterial genus (Fig. 1c,d). DMM1 sputum microbiota were primarily dominated by *Streptococcus*, DMM2 by *Prevotella*, DMM3 by *Haemophilus* and DMM4 by *Moraxella*. Indeed, using dominant taxon identity as a classifier for each sample increased our capacity to explain the observed variance in sputum microbiota composition (PERMANOVA, R^2^ = 0.492, p < 0.001), and indicated that dominant taxon classification was broadly consistent with the DMM clusters identified.

### Association of sputum microbiota with HIV-associated chronic lung disease

Using dominant taxon as a classifier, we noted that subjects with a *Haemophilus-, Moraxella-* or *Neisseria*-dominated sputum microbiota (*HMN* group; n = 35) were more likely to be associated with CLD relative to those with either a *Streptococcus* or *Prevotella*-dominated airway microbiota (*SP* group, n = 116; RR = 1.50, p = 0.024; Table 2). Time on ART (5.0 vs. 4.7 years, p = 0.45), CD4+ T-cell count (717 vs. 727 cells/µL, p = 0.66), and proportion virologically suppressed (66% (n = 23/35) vs. 71% (n = 79/111); p = 0.69) did not differ statistically between the HMN and SP groups. The strongest association with chronic lung disease occurred among HIV-infected children with a *Neisseria*-dominated sputum microbiota, though these individuals were rare in our cohort.

**Table 2:** Distinctly dominated sputum microbiota associate with risk of HIV-associated chronic lung disease.

	RR	p value
*Haemophilus/Moraxella/Neisseria *vs.* Streptococcus/Prevotella*	**1.500**	**0.024**
*Haemophilus* vs. *Moraxella*	1.154	0.746
*Haemophilus* vs. *Neisseria*	**0.577**	**0.001**
*Haemophilus* vs. *Streptococcus*	1.435	0.081
*Haemophilus* vs. *Prevotella*	1.500	0.297
*Moraxella* vs. *Neisseria*	0.500	0.090
*Moraxella* vs. *Streptococcus*	1.244	0.608
*Moraxella* vs. *Prevotella*	1.300	0.626
*Neisseria* vs. *Streptococcus*	**2.488**	** < 0.001**
*Neisseria* vs. *Prevotella*	**2.600**	**0.007**
*Streptococcus* vs *Prevotella*	1.045	0.905

Values in bold were considered statistically significant at *p* ≤ 0.05.

### *HMN* sputum microbiota induce epithelial cell inflammation

**Figure 2:**
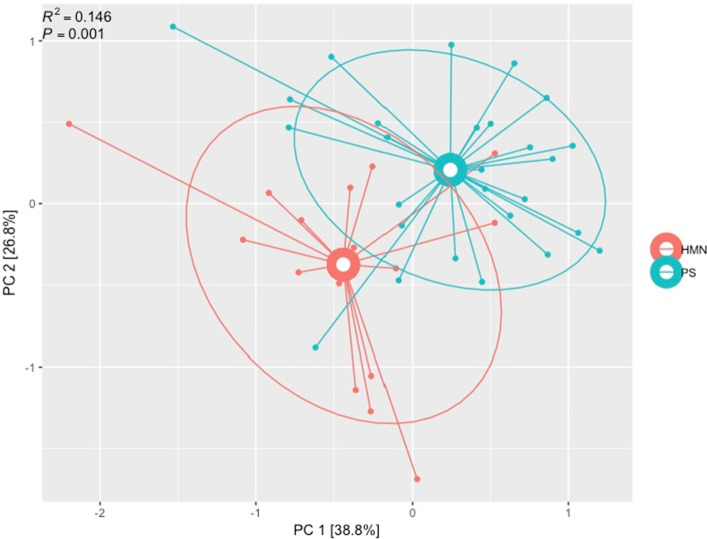
PCoA plot of epithelial gene expression profiles induced following exposure of cultured epithelial cells to sterile products of the HMN or SP patient sputum. Data indicates that sputum from the *Haemophilus*-*Moraxella*-*Neisseria* (*HMN*) group induces a significantly distinct epithelial gene expression response from that induced by the sterile sputum products of the *Prevotella*-*Streptococcus* (*PS*) group.

We hypothesized that increased risk of CLD associated with distinct sputum microbiota is due to enhanced inflammatory activity by CLD-associated airway microbiota. Because we had observed that the *HMN* group was at significantly higher risk of CLD compared with the *SP* group, we analyzed a representative sample of 50 (of 151) sputum samples from children with HIV-CLD belonging to the *HMN* (n = 19) or *SP* [*S* (n = 18), *P* (n = 13)] groups for their capacity to promote epithelial inflammation. Human airway epithelial A549 cells were treated with cell-free sputum extracts for 24 h. Data from A549 exposure to sterile sputum fluid (SSF) was considered in our analyses if > 60% cell viability was observed following 24 h of SSF treatment. The majority of SSF exposures (n = 45/50) resulted in > 60% viability (Supplemental Figure 3). Q-PCR based expression analysis of 9 genes involved in epithelial inflammatory processes (IL8, IL33, IL1β, CXCL10, TGFβ), barrier function (Occludin, E-cadherin, Muc5AC), and response to microbes (TLR9) were assessed (Supplemental Figure 4). Principal coordinate analysis indicated that the expression profile of these 9 genes was significantly distinct based on whether sputum samples belonged to the *HMN or SP* groups (PERMANOVA, R^2^ = 0.16, p = 0.001; Fig. 2)*.* This indicates that the products of these distinct sputum microbiota differentially interact with airway epithelia to induce distinct responses.

**Figure 3:**
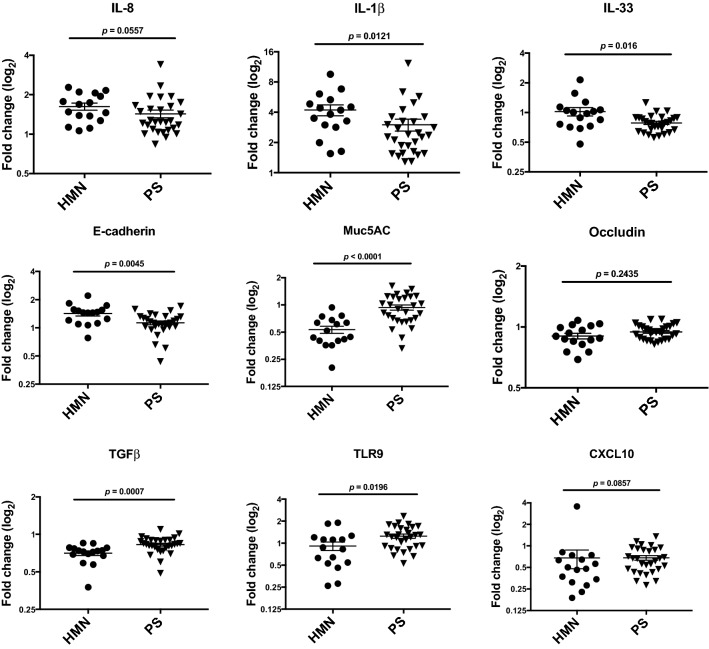
Comparative analysis of epithelial gene expression induced following exposure of cultured epithelial cells to sterile products of *Haemophilus*-*Moraxella*-*Neisseria* (*HMN*) or *Prevotella*-*Streptococcus* (*PS*) sputum.

More specifically, we noted that sputum products of *HMN* microbiota (compared with those of the *SP* group) induced significantly increased expression of IL-1β (p = 0.01), IL-33 (p = 0.01) and E-cadherin (0.004), which play a role in cell to cell adherence, as well as decreased expression of Muc5AC (p < 0.0001) associated with mucin secretion, TGFβ (p = 0.0007) which is considered anti-inflammatory, and TRL9 (p = 0.01) which senses and responds to microbial DNA (Fig. 3).

## Discussion

HIV-associated chronic lung disease affects up to one-third of children and adolescents on long-term ART in sub-Saharan Africa^3^, but little is understood about its pathophysiology. To our knowledge, our study is the first to apply culture-independent 16S rRNA gene sequencing to sputum to investigate molecular mechanisms underlying HIV-CLD pathogenesis. We identified four compositionally distinct microbiota profiles in the sputum of older children with HIV consisting of microbiota dominated by *Streptococcus*, *Haemophilus*, *Prevotella*, *Moraxella,* or *Neisseria*. Children with *Haemophilus*-, *Moraxella*-, or *Neisseria*-dominated sputum microbiota (HMN group) were 1.5 times more likely to have CLD compared to children dominated by *Streptococcus* or *Prevotella (SP* group*)*. In addition, cell-free products of *HMN* sputum induced a distinct epithelial gene expression signature characterized by increased expression of pro-inflammatory cytokines and decreased expression of Muc5AC. Muc5AC stimulates anti-inflammatory TGFβ and produces mucin-5AC^10^, a gel-forming glycoprotein responsible for mucosal protection from infection and chemical damage, suggesting that airway colonization by microbiota in the HNM group induce a higher degree of inflammation. Therefore, if confirmed, our findings suggest that interventions targeting modulation of lung microbiota could have important treatment implications for CLD.

Microbiota dominated by *Neisseria, Moraxella,* or *Haemophilus* are associated with chronic respiratory diseases including asthma, COPD, and chronic rhinosinusitis among HIV-uninfected adult and pediatric populations^6,11–15^. In contrast, these findings have not consistently been supported in HIV-positive populations, where *Streptococcus* and *Prevotella* dominance has been associated with pulmonary inflammation, COPD, and short- and intermediate-term mortality among HIV-positive Ugandan adults hospitalized with acute pneumonia^9,16,17^. The association of *Streptococcus* and *Prevotella* microbiota with a lower relative risk of pediatric HIV-CLD in our study could be explained by three factors. First, a distinct bacterial composition among children due to less lifetime ART exposure, less immune system maturation and expected microbiota establishment due to peri-natal acquisition of HIV, more frequent antibiotic use, and differing response to vaccinations and environmental exposures^15^. Second, 16S rRNA sequencing currently classifies dominant microbes to the genus level, and we cannot rule out genomic differences in species or strains within these genera as accounting for differences in disease associations. Third, a microbiota’s functional role in host immune modulation is driven by microbe-microbe and microbe-host interactions and resulting antigenic and metabolic products, rather than the relative abundance of any individual taxa^9,18,19^. Therefore, *Haemophilus, Moraxella, Neisseria*, *Streptococcus* and *Prevotella* all represent a spectrum of pathogenic potential in HIV-positive children, with *HMN* as a group exerting enhanced inflammatory effects and conferring an increased relative risk of HIV-CLD.

Microbe-mediated modulation of epithelial gene expression provides a critical mechanistic link between *HMN* community composition and HIV-CLD pathogenesis. We demonstrate that the *HMN* group is associated with significantly higher levels of pro-inflammatory cytokines compared to *Streptococcus*- or *Prevotella*-dominated sputum samples. These include IL-1β, an important epithelial cell-to-cell signal that stimulates macrophage accumulation in early infection^20^, IL-33, a known driver of type-2 immune response during infection^21^, and E-cadherin, a protein important for epithelial barrier integrity, which has been shown to be down-regulated with increased IL-33^21^.

Our findings suggest that *Moraxella* and *Haemophilus* may be principal drivers of airway inflammation in HIV-CLD. Molecular mechanisms of immune modulation by *Moraxella catarrhalis (M. catarrhalis)* and non-capsulated *Haemophilus influenza* (NHi) are well-established in COPD^22–24^. Certain groups of Moraxella (e.g., *Moraxella catarrhalis*) are known lung pathogens^25^ able to invade host epithelial cells and survive as intracellular pathogens. *Moraxella* stimulates an EGFR-mediated NF-kB and ERK pathway activation, leading to neutrophil infiltration in lung epithelial cells through an increase in IL-8 production^22,26^. NHi has also been known to invade host epithelial cells, escape host immune response, form biofilms, and increase severity and duration of co-occurring viral infections^18,23,24^. It has been implicated in initiating a robust TLR-mediated cellular immune response via NF-kB activation, increasing IL-1β and IL-8 production and subsequent neutrophil infiltration into the lung^24,26,27^. In addition, *Moraxella, Haemophilus* and *Neisseria* all contain homologous lactoferrin-binding and transferrin-binding proteins that allow them to sequester local iron sources to outgrow other commensals and form polymicrobial biofilms that are associated with COPD exacerbations^28–30^. Though extrapolations from pro-inflammatory mechanisms of *M. catarrhalis* and NHi in COPD remain theoretical, they provide biological plausibility to our hypothesis.

Our data further illustrate decreased expression of TGF-β and TLR9 in the *HMN* group. In HIV-infected patients, TGF-β functions in various ways as an anti-inflammatory agent in both innate immunity, such as suppressing survival and proliferation of B lymphocytes, and adaptive immunity, such as inhibiting mTOR-mediated NF-κB activation^31^. *Moraxella* and *Haemophlius*-mediated NF-κβ activation and resultant epithelial inflammation, as detailed above, likely overwhelms TGF-β function in *HMN* subjects. TRL9 similarly participates in recognizing bacterial and viral DNA at the lung epithelial surface, in attenuating pro-inflammatory innate and adaptive immune responses, and in tissue homeostasis^32^. A blunting of these functions likely contributes to increased pro-inflammatory cytokines in *HMN* subjects.

Our study has several potential limitations. First, our study was cross-sectional and unable to determine the temporal relationship of the oral microbiome to development or progression of clinical chronic lung disease in HIV-infected older children. Further, the effect sizes of examined variables to the microbiome were relatively small, indicating that other factors might have contributed to the microbiome alterations and development of chronic lung disease. Second, our in vitro epithelial gene expression analysis provided a plausible explanation of lung microbe function in mucosal immune modulation, but we did not carry this forward to in vivo verification. Third, HRCT was only performed for participants with findings noted on plain chest radiograph; since computed tomography is much more sensitive for lung pathology, our prevalence estimate for radiographic abnormality likely represents a lower bound. Fourth, we did not include a control group of children without HIV infection. Relative to adults without HIV infection, adults with advanced HIV infection demonstrate decreased oral microbial α diversity^17,33^, and adults with well-controlled HIV infection demonstrate increases in relative abundance of *Veillonella*, *Streptococcus*, and *Lactobacillus*^34^. The degree to which these findings extend to HIV-uninfected children and adolescents living in sub-Saharan Africa is unknown. Finally, we utilized induced sputum rather than BAL, often considered the gold standard procedure for sampling of the lower airway microbiome^35^. However, due to subclinical microaspiration and bronchial mucosa migration, lung bacterial communities largely resemble those in the mouth^36,37^ and the most abundant pathogen in sputum generally reflects the predominant taxa identified in BAL^38^. Further, the cystic fibrosis literature supports sputum induction as a non-invasive surrogate for bronchoalveolar lavage^39^.

In conclusion, we determined through culture-independent 16S rRNA sequencing that an oral microbiota profile dominated by *Haemophilus*-, *Moraxella*-, and *Neisseria*, relative to one dominated by *Streptococcus* or *Prevotella,* was associated with chronic lung disease among older children with well-controlled HIV infection. We demonstrated the plausibility of this finding mechanistically with epithelial cell gene expression experiments. Prospective longitudinal studies that demonstrate an in vivo microbial or metabolic change preceding comprehensive chronic lung disease phenotypes are now needed.

## Materials and methods

### Study subjects

We undertook a cross-sectional study of HIV-infected children aged 6–16 years receiving routine outpatient HIV care at the Harare Central Hospital in Harare, Zimbabwe, between September 2014 and June 2015. For logistical ease, the first five eligible children in the outpatient HIV clinic were consecutively enrolled every weekday. Recruitment was limited to those established on ART for ≥ 6 months. Children with signs and symptoms of acute respiratory tract infection, defined as new or worsening respiratory symptoms, fever, night sweats, or other systemic symptoms in the past 1 week, were excluded. Written informed consent from guardians and verbal consent from children was obtained prior to enrollment (University of California, San Francisco Human Research Protection Program #14-13648).

### Data collection

#### Clinical investigations

Methods pertaining to data collection have been previously published^2^. Respiratory symptoms were assessed by nurse-administered questionnaire. Spirometry was performed according to American Thoracic Society (ATS) standards using EasyOne World spirometers (ndd Medical Technologies, Inc., Andover, Massachusetts, USA)^40^. Only spirometry data that met ATS quality criteria was used for analysis. All subjects underwent chest radiography and those with any radiographic abnormality on plain film underwent chest high-resolution computed tomography (HRCT). The radiographic findings from this cohort have been previously published^41^. HIV-CLD was operationally defined as (1) reduced FEV1:FVC ratio on spirometry; (2) chronic cough (regardless of character) and/or dyspnea, defined as presence on most days for ≥ 4 weeks; or (3) abnormal HRCT (bronchial wall thickening, mosaic attenuation, and bronchiectasis)^41^.

#### Laboratory investigations

Blood samples were collected to establish CD4 counts (PIMA platform; Alere, Waltham, MA), and HIV viral load (COBAS Ampliprep/Taqman 48; Roche, Pleasanton, CA). Induced sputum samples were collected using nebulized hypertonic saline. Samples were examined by Ziehl-Neelson stain microscopy and a single mycobacterial culture was performed on Lowenstein-Jensen media.

#### 16s rRNA gene profiling

Genomic DNA was extracted from 500ul of raw sputum consisting of sputum plugs and liquid via mechanical lysis using a modified cetyltrimethylammonium bromide (CTAB) buffer method as we have previously described^42^. DTT was not used during the processing of sputum because of possibility that the 30-min incubation at 37 °C necessary for this protocol may alter sputum microbiota profiles. The V4 region of the 16S rRNA gene was amplified in triplicate PCR reactions as previously described^42^. Purified amplicons were pooled and sequenced on a 151 bp × 151 bp paired end sequencing run on the Illumina Nextseq500 platform (San Diego, California) containing 40% phiX. Negative controls for extraction (extraction buffers), 16S rRNA amplification (no template control) and sequencing (concentrated amplicon from negative controls) were built into the protocol^43^.

#### Data analysis

Sequence data was converted to fastq format using bcl2fastq v2.16.0.10 and paired sequencing reads with a 25 bp minimum overlap were merged using FLASH v1.2.11. Merged reads were demultiplexed in the absence of quality filtering using QIIME (Quantitative Insights into Microbial Ecology, v1.9.1) followed by quality filtering using the USEARCH fastq filter (v7.0.1001) to remove reads having > 2 expected errors. Quality filtered reads were dereplicated at 100% identity, clustered at 97% sequence identity into operational taxonomic units (OTUs), had chimeras removed, and were mapped back to the resulting OTUs using USEARCH v8.0.1623. Taxonomy was assigned using the Greengenes database (May 2013). OTUs were filtered by (1) removing any OTU with the taxonomic classification of a known common contaminant present in more than half of the Negative Extraction Controls, (2) subtracting the maximum read counts found in any single remaining OTU present in Negative Extraction Controls from all samples, and (3) removing any remaining OTU that had a total read count across all samples less than 1/1000th of a percent of the total read counts across all samples. Finally, sequencing reads were rarefied to 73,265 reads/sample as described previously^42^. Dominant taxon was defined as the taxon that exhibited the highest relative abundance within a given sample.

#### Respiratory epithelial gene expression assay

Sputum samples for epithelial assays were randomly selected from HMN or SP groups. Individual frozen sputum samples were heated to 37 °C for 10 min. Samples were vortexed for 60 s to fully resuspend the contents prior to microcentrifugation at 14,000 rpm for 10 min at room temperature. Samples were filter sterilized by first filtering the supernatant through Whatman 0.45 μm PVDF Mini-UniPrep filter (Cat. No. US203NPEAQU, GE Healthcare Life Sciences) followed by filtration through a 0.22 μm filter (Cat. No. US203NPUAQU) to obtain sterile sputum fluid (SSF).

As in prior investigations^44^, human airway epithelial A549 cells were seeded at a confluent density in 96-well plates in fresh RPMI 1640 medium (Gibco, Thermo Fisher Scientific) supplemented with 10% heat-inactivated Fetal Calf Serum (Cat. No. 9871-5244, USA Scientific), 100 U ml^−1^ penicillin–streptomycin (Cat. No. 10378016, Life Technologies, Carlsbad, CA) and incubated at 37 °C under conditions of 5% CO_2_ for 24 h. Spent culture media was exchanged for fresh media containing SSF (1 in 4 dilution SSF in RPMI media). Triplicate exposures for each sputum fluid sample was performed and cells were re-incubated for 24 h. Total RNA was isolated with RNAqueous-Micro Kit (Cat. No. AM1931, Thermo Fisher Scientific) and digested with DNase I. First-strand cDNA was synthesized using 200 ng RNA with High-Capacity RNA-to-cDNA Kit (Cat. No. 4387406, Thermo Fisher Scientific) according to the manufacturer’s instructions.

Real-time quantitative-PCR (qPCR) was performed in triplicate using SYBR Green master mix (Cat. No. 4368577, Life Technologies) in the QuantStudio 6 Flex Real-Time PCR System (Life Technologies, Carlsbad, CA)^44^. The PCR reaction conditions were as follows: 50 °C for 2 min, 95 °C for 10 min (1 cycle); 95 °C for 15 s and 60 °C for 1 min (40 cycles); and a final melting curve cycle of 95 °C for 15 s, 60 °C for 1 min and 95 °C for 15 s. Target gene expression was normalized to β-actin expression followed by ∆*CT* subtraction of media-treated control to calculate the 2^−∆∆*CT*^ (where *C*_*T*_ is threshold cycle) value. Forward and reverse primers used for qPCR analysis of target genes are listed in Supplemental Table 1.

### Data analysis

Our primary outcome was to determine the association of sputum bacterial community composition (i.e., microbiota) with HIV-CLD. We identified clusters of subjects based on bacterial community composition using Dirichlet Multinomial Mixture (DMM) models, an unsupervised Bayesian approach as previously implemented^42^. The best fitting DMM model was determined using the Laplace approximation. Weighted UniFrac distance matrixes were calculated using QIIME to assess compositional dissimilarity between samples and visualized using PCoA plots. Permutational multivariate analysis of variance (PERMANOVA) was performed using the adonis2 function in the *vegan R* package (version 2.5-3) to assess factors significantly explaining variation in microbiota β–diversity. Differences in covariates between each DMM and all other DMMs, and for DMM groups 1/2 vs. DMM groups 3/4 were determined using logistic regression. P-values were adjusted for multiple comparisons using Benjamini–Hochberg false discovery rate correction. CD4+ results were treated as parametric data; other continuous variables were nonparametric, where central tendency was reported by the median and interquartile range (IQR) and the Mann–Whitney U test was used for equivalence testing between groups. Frequencies of categorical data were compared by chi-squared test, and results were considered statistically significant at p ≤ 0.05 (R Core Team, version 3.5.1/3, https://www.R-project.org/).

### Methods statement

All methods in this manuscript were carried out in accordance with relevant guidelines and regulations. Ethical approval was obtained from the University of California, San Francisco Human Research Protection Program (#14-13648).

## Supplementary information

Supplementary Information.
