# Supplementary material for: Distinct lung microbiota associate with HIV-associated chronic lung disease in children
Source: Sci Rep. 2020 Sep 30;10:16186. doi: 10.1038/s41598-020-73085-1 (PMC7527458; doi:10.1038/s41598-020-73085-1)
Supplement: Supplementary file 1 — Supplementary Information. [file 41598_2020_73085_MOESM1_ESM.docx]

**ONLINE DATA SUPPLMENETAL TEXT**

**Distinct lung microbiota associate with HIV-associated chronic lung disease in children**

*Sudha Bhadriraju, MD, MPH,^1^ *Douglas W. Fadrosh, M.S.,^2^ Meera K. Shenoy, PhD, ^2^ Din L. Lin, PhD,^2^ Kole V. Lynch, B.S.,^2^ Kathryn McCauley M.S.^2^ Rashida A. Ferrand, MD, MSc, PhD,^3,4^ Edith Majonga, MD, PhD,^3,4^ Grace McHugh, MBBCh,^3^ Laurence Huang, MD,^1^ *Susan V. Lynch, PhD,^2^ *John Z. Metcalfe, MD, PhD, MPH ^1^

^1^ Division of Pulmonary and Critical Care Medicine, San Francisco General Hospital and Trauma Center, University of California San Francisco, San Francisco, USA; ^2^ Division of Gastroenterology, Department of Medicine, University of California, San Francisco, San Francisco, USA; ^3^ Biomedical Research and Training Institute, Harare, Zimbabwe; ^4^ London School of Hygiene and Tropical Medicine, London, UK

*Contributed equally

**Tables**

**Supplemental Table 1. PCR primers for gene expression analysis**

| Gene | Forward primer | Reverse primer |
| --- | --- | --- |
| IL-1β | AAACAGATGAAGTGCTCCTTCCAGG | TGGAGAACACCACTTGTTGCTCCA |
| IL-8 | ACTGAGAGTGATTGAGAGTGGAC | AACCCTCTGCACCCAGTTTTC |
| IL-33 | TCAGGTGACGGTGTTGATGG | GGAGCTCCACAGAGTGTTCC |
| TGFβ | GCGTGCTAATGGTGGAAAC | CGGTGACATCAAAAGATAACCAC |
| E-cadherin | TCATGAGTGTCCCCCGGTAT | GTCAGTATCAGCCGCTTCAGAT |
| Occludin | GATGAGCAGCCCCCCAAT | GGTGAAGGCACGTCCTGTGT |
| TLR9 | TTCCTCTATTCTCTGAGCCG | GTAGGAAGGCAGGCAAGGTA |
| CXCL10 | CCAGAATCGAAGGCCATCAA | CATTTCCTTGCTAACTGCTTTCAG |
| Muc5AC | TACTCCACAGACTGCACCAACTG | CGTGTATTGCTTCCCGTCAA |
| β-Actin | AAGATGACCCAGATCATGTTTGAGACC | AGCCAGTCCAGACGCAGGAT |

**Supplemental Table 2. Characteristics of study participants**

|  | **Participants with microbiome sample (n=146)** | **Participants without microbiome sample (n=56)** | **p-value** |
| --- | --- | --- | --- |
| Age (years), mean (SD) | 10.8 (2.5) | 9.8 (2.7) | 0.01* |
| Female sex, n (%) | 66 (45.2%) | 31 (55.4%) | 0.26 |
| Age at diagnosis (years),  mean (SD) | 5.3 (2.8) | 4.4 (3.0) | 0.01* |
| Duration of ART at recruitment (years), mean (SD) | 4.7 (2.6) | 5.1 (2.6) | 0.56 |
| CD4+ at enrollment (cells/uL), mean (SD) | 511.5 (505.05) | 385 (338.6) | 0.37 |
| Viral load at enrollment (copies/mL), median (IQR)† | 19 (313) | 19 (28) | 0.21 |
| Proportion virally suppressed (<75 copies/mL) | 69.9% | 80.4% | 0.2 |
| Previous TB treatment‡ | 56 (38.6%) | 20 (35.7%) | 0.83 |
| Treated with >1 course of antibiotics in the past 12 months‡ | 31 (21.2%) | 16 (28.6%) | 0.36 |
| Stunted growth‡§ | 51 (34.9%) | 21 (38.2%) | 0.86 |
| Bronchiectasis on HRCT‡ | 22 (35.4%) | 6 (35.3%) | 1 |

*Definition of Abbreviations:* SD=standard deviation; CLD=chronic lung disease; TB=tuberculosis; HRCT=high resolution chest computed tomography

*p<0.05 = significant; † Wilcoxan-rank sum test; ‡ Chi-square test; § ”Height for age" value <two standard deviations of the WHO Child Growth Standards^1^.

**Figures**

**Supplemental Figure 1. Flow chart for sputum sample processing**

**Supplemental Figure 2. Laplace approximation of distinct microbiota structures**

**Supplemental Figure 3. Epithelial cell viability**


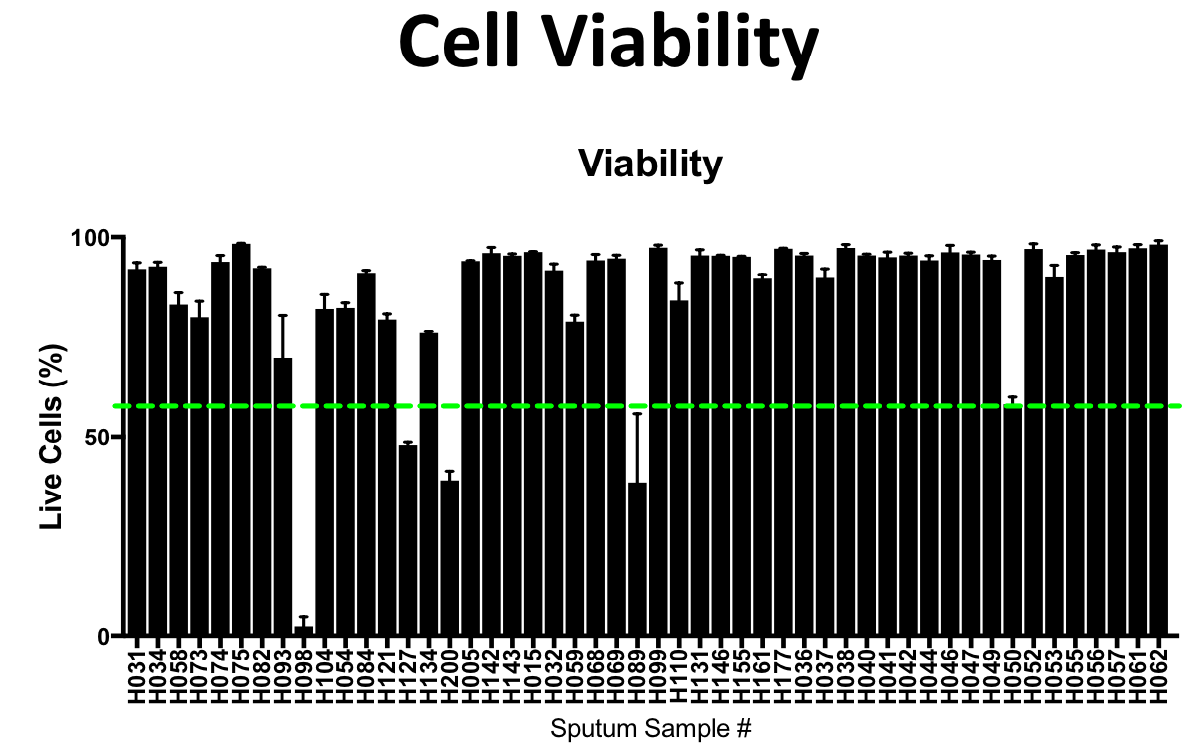


**Supplemental Figure 4. Heatmap of gene expression stratified by microbiota composition**


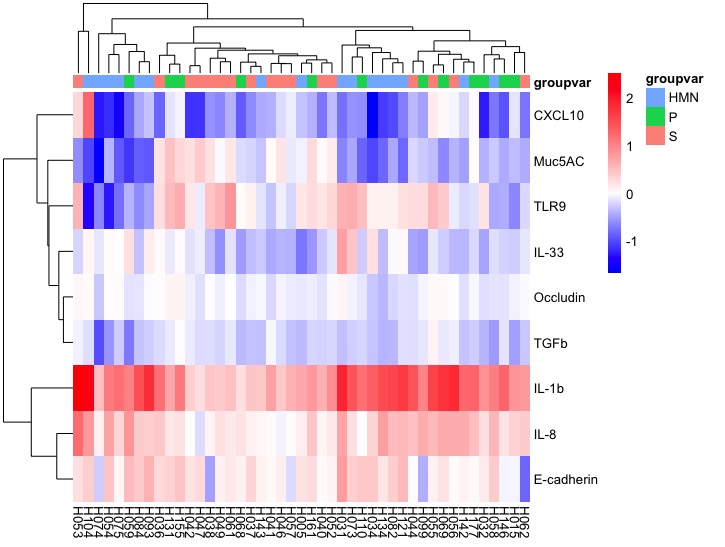


References

1 World Health Organization. World Health Organization Growth Reference, Height-for-age, 5-19 years. (2017).
